# COVID-19 Pneumonia in Vaccinated Population: A Six Clinical and Radiological Case Series

**DOI:** 10.3390/medicina57090891

**Published:** 2021-08-27

**Authors:** Barbara Brogna, Elio Bignardi, Claudia Brogna, Chiara Capasso, Giuliano Gagliardi, Alberigo Martino, Lanfranco Aquilino Musto

**Affiliations:** 1 Department of Radiology, San Giuseppe Moscati Hospital, Contrada Amoretta, 83100 Avellino, Italy; giuliano.gagliardi@hotmail.it (G.G.); musto.lanfranco@gmail.com (L.A.M.); 2 Radiology Unit, “Cotugno Hospital”, Naples, Via Quagliariello 54, 80131 Naples, Italy; dr.eliobignardi@alice.it; 3 Pediatric Neurology Unit, Fondazione Policlinico Universitario “A. Gemelli”, IRCSS, Università Cattolica del Sacro Cuore, 00168 Rome, Italy; claudiabrogna@yahoo.it; 4 Neuropsychiatric Unit, ASL Avellino, Via Degli Imbimbo 10/12, 83100 Avellino, Italy; 5 Pharmacology Department, “Frangipane” Hospital, ASL Avellino, Via V. Emanuele, 83031 Ariano Irpino, Italy; chiara.ftvz@gmail.com; 6 Radiology Unit, “Frangipane” Hospital, ASL Avellino, Via V. Emanuele, 83031 Ariano Irpino, Italy; albmart@libero.it

**Keywords:** SARS-CoV-2, COVID-19 vaccines, COVID-19 pneumonia, immunocompromised state, case reports

## Abstract

Severe Acute Respiratory Syndrome Coronavirus-2 (SARS-CoV-2) and its related disease (COVID-19) continue to represent a challenge for humans. To date, vaccination programs have represented an opportunity to navigate the pandemic. However, the advent of new genetic COVID-19 variants has increased more attention representing a worrying threat not only for not vaccinated but also for vaccinated people as virus infections have been shown also in the last ones. Herein, we report different clinical cases and radiological findings of COVID-19 pneumonia in six fully vaccinated patients. Two patients had a history of Rituximab therapy for follicular lymphoma and with persistent positivity for SARS-CoV-2 on nasopharyngeal/oropharyngeal (NP/OP) swabs and with moderate pneumonia on the chest computed tomography (CT). One patient who resulted to be positive to delta variant 8 days after the second vaccination dose, died shortly after. Two patients were hospitalized due to the worsening of fever and dyspnea in presence of mild pneumonia on CT. In one patient mild pneumonia was found on the chest-CT performed after a lipothymic episode associated with chest pain and positive NP/OP swab tested for SARS-CoV-2. These data suggested that in fully vaccinated people, caution should be preserved, and the use of masks and social distancing should be continued in all closed environments. However, further clinical trials should be done to better understand how various factors can influence vaccine immunogenicity as the presence of virus mutations, age factors, and the presence of an immunocompromised state.

## 1. Introduction

The pandemic caused by the Severe Acute Respiratory Syndrome Coronavirus-2 (SARS-CoV-2) and its related disease (COVID-19) represents a major challenge for humans. To date, COVID-19 has caused more than 3 million deaths, leading to economic troubles in several countries [1,2,3,4].

COVID-19 has a multisystem involvement affecting not only the lungs but also the cardiovascular, nervous, and gastrointestinal systems. In adults, and especially in children, COVID-19 has led to a multisystem inflammatory syndrome [1,2,3,4].

Different means of virus transmission have been reported. COVID-19 transmission can occur through direct contact from one person to another by coughing, sneezing, and inhaling infected droplets, or by contact with oral and nasal mucosa or conjunctival mucosa after touching an infected surface [5]. COVID-19 transmission has also been reported to follow the fecal-oral route [3].

Asymptomatic individuals with COVID-19 infection can be a source for the virus transmission [6].

To date, reverse-transcription polymerase chain reaction (RT-PCR) testing is considered to be the gold standard tool to diagnose or screen for COVID-19. Chest imaging is currently indicated in suspected cases to support rapid medical triage in the presence of high pretest probability [7]. CT is mainly useful in suspected cases of COVID-19 pneumonia with the presence of comorbidities [8,9]. Different pharmacological approaches, including antibiotics, corticosteroids, and immunomodulant therapies, have been used with different gradings of clinical responses depending on the severity of the disease [10]. In addition, monoclonal antibodies, recently approved by the Food and Drug Administration (FDA), have been used to treat symptomatic people in both the adult and pediatric populations in an emergency stage [11].

Vaccination against SARS-CoV-2 is considered the most efficient solution. Vaccination programs have been approved worldwide, aiming to reduce morbidity and mortality, especially in frail people. Today, there are several vaccines against the novel coronavirus. Some of these vaccines have been quickly approved by the European Medicines Agency (EMA) and FDA, including the Pfizer mRNABNT162b2, Moderna mRNA -1273, Vaxzevria (ChAdOx1-S; AstraZeneca), and Johnson and Johnson vaccines [12,13,14]. These vaccines have demonstrated high efficacy in clinical trials [15,16]. However, concerns remain regarding the rapid spread of new virus variants and the increasing number of new cases in countries with high percentages of vaccinated people [17,18,19,20]. We report six different cases of COVID-19 pneumonia in fully vaccinated patients.

## 2. Case #1

A 71-year-old man with a history of hypertension received a full dose of the Vaxzevria vaccine (ChAdOx1-S; AstraZeneca) (first dose on 1 April and second dose on 24 June). Three days after the second dose, he reported cough and fever. However, after 8 days (on 2 July), he visited the emergency room of our hospital due to the worsening of symptoms, including fever and dyspnea.

On admission, the patient’s nasopharyngeal/oropharyngeal (NP/OP) swab tested positive with the detection of the Delta variant. On laboratory examination, he showed a lymphocyte count in the lower range (1100/µL; normal value range 800–5000/µL), an elevation of the D-Dimer level (4810 ng/mL; normal value < 250 ng/mL), and a mild elevation of the C-reactive Protein (CRP, 2.12 mg/dL; normal value < 0.5). The other laboratory values were within the normal range. On admission, the Saturation oxygen level (SO2) was at 88% with PO2/FiO2 290.

**Figure 1:**
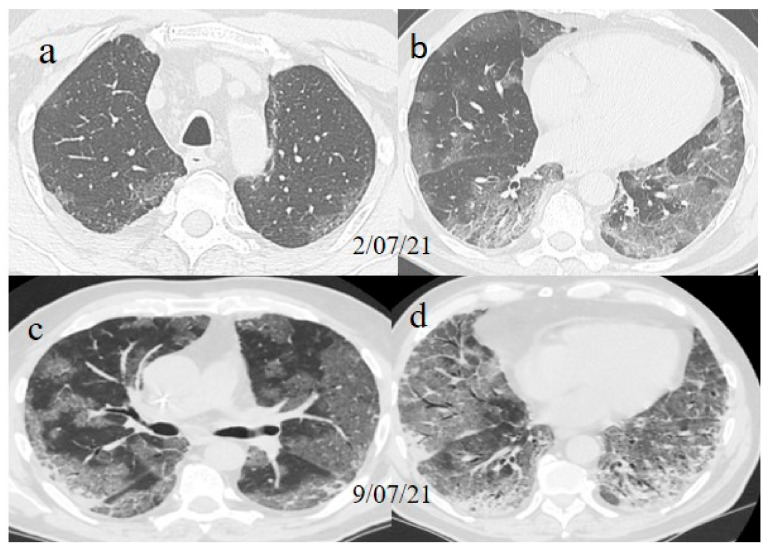
(**a**,**b**) The basal CT with GGO in a typical peripheral and posterior distribution of COVID-19 pneumonia with a CT-SS of 13/20. (**c**,**d**) The chest CT performed 1 week later, showing more extensive pneumonia with a CT-SS of 16/20.

A chest Computed tomography (CT) performed in the emergency department showed a typical central and peripheral distribution of ground-glass opacity (GGO) COVID-19 pneumonia. The CT-SS score [21] showed a value of 13/20 (Figure 1).

The serology, performed with an immunoassay (LiaisonXL), confirmed the presence of SARS-CoV-2 S1/S2 IgM associated with SARS-CoV-2 anti-spike IgG (2080 BAU/mL) (<33.80 BAU/mL: Absent) (>33.80 BAU/mL: presence), which was related to the previous vaccination.

Treatment with dexamethasone (6mg once daily with intravenous administration for 10 days) and conventional oxygen therapy was started, together with low molecular weight heparin (LMWE) at 4000 IU (twice daily with a subcutaneous administration for 10 days).

One week later (on 9 July), another chest CT was performed due to the worsening of the patient’s symptoms with SO2 at 76% and PO2/FiO2 190.

The chest CT showed extended pneumonia, with an evolution in a crazy paving pattern and consolidation areas (CT-SS 16/20) (Figure 1). The patient died 5 days later.

## 3. Case #2

**Figure 2:**
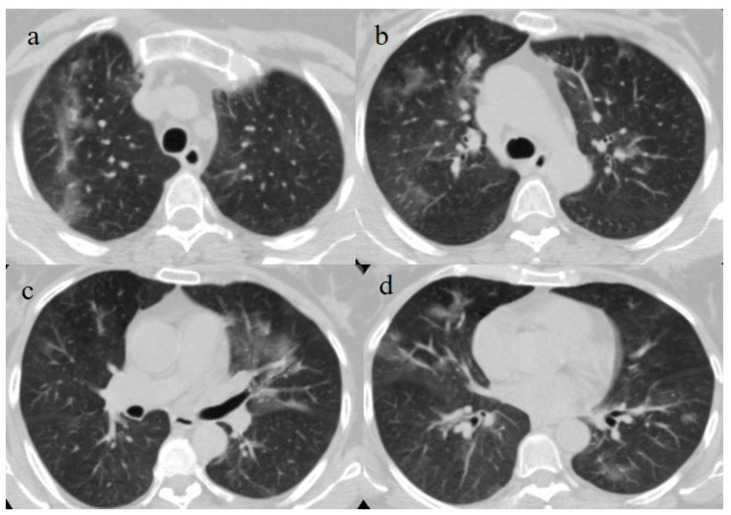
Chest CT performed in the emergency department on 7 June, showing GGO areas with a peripheral and central distribution in a typical presentation of COVID-19 pneumonia in a multifocal involvement. (**a**,**b**) GGO areas in the superior lobes, (**c**) the middle and left superior lobes, and (**d**) the inferior lobes.

A 51-year-old woman with a history of follicular lymphoma in remission in September 2020, and with a previous SARS-CoV-2 infection in November 2020, received only one dose (according to the current evidence) [22,23] of the Pfizer mRNABNT162b vaccine on 7 April 2021. The patient had been undergoing treatment with rituximab since September 2020. However, the patient never developed an immune response to SARS-CoV-2 as confirmed by serology, and she had shown a persistent mild positivity on RT-PCR for SARS-CoV-2 since the day of vaccination. A few days after the vaccination, her clinical condition worsened, with dyspnea, fever, and a SO2 of 96%. One week after the vaccination, she visited the emergency room of a tertiary hospital and underwent a chest computed tomography (CT). The chest CT showed multilobe areas of ground glass (GGO) in a peripheral and central distribution in the acute phase. The patient’s RT-PCR for SARS-CoV-2 continued to be positive. After 2 months, on 7 June, she visited the emergency room of our hospital for the worsening of dyspnea and fever. On admission, another NP/OP swab tested positive for SARS-CoV-2 without the detection of any variants. Her SO2 was at 90%. A chest CT was performed in the emergency department, showing GGO in a peripheral and central distribution with a CT-SS of 9/20 (Figure 2).

On laboratory examination, she showed a low level of hemoglobin (Hb) (9.5 g/dL; normal range 13.0–16.5), a lower white blood count (2400/µL; normal value range 4300–10,800/µL), a mild elevation of the D-Dimer level (1.43 mg/L; normal value < 0.5), an elevation of the C-reactive Protein (CRP, 11.41 mg/dL; normal value < 0.5) and an elevation of lactate dehydrogenase enzyme (LDH, 360 IU/L; normal value 125–220). The other laboratory values were within the normal range. Serology showed the absence of anti-spike IgG despite the previous vaccination. Treatment with dexamethasone (4 mg once daily with intravenous administration for 10 days) and conventional oxygen therapy was started (1 L/min) with SO2 at 97%, together with low molecular weight heparin (LMWE) 4000 IU (twice daily with a subcutaneous administration for 10 days).

**Figure 3:**
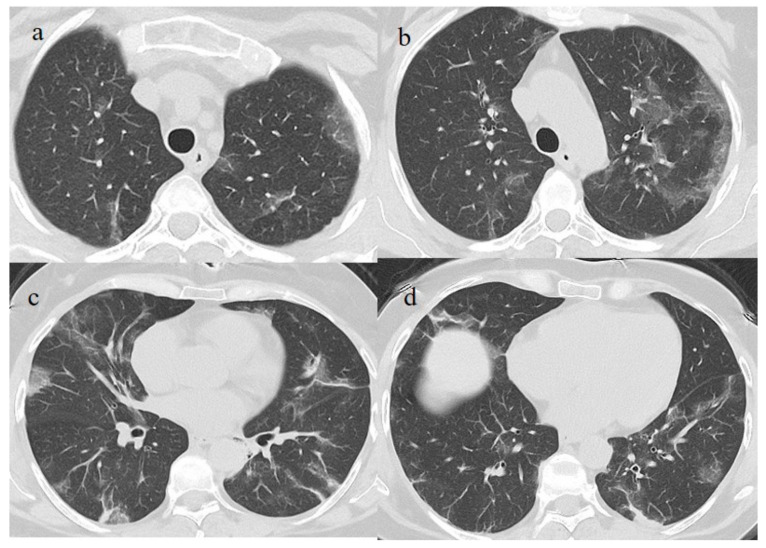
Chest CT performed on 17 June. (**a**,**b**) Some new GGO areas in the left superior lobe with the disappearance of the GGO areas in the superior lobe. (**c**,**d**) An evolution in crazy paving and the consolidation of some previous GGO in the middle and inferior lobes.

The patient’s NP/OP swabs continued to test positive for SARS-CoV-2 on 11 and 15 June. Another chest CT was repeated on 17 June, which showed an evolution of some GGO areas in consolidative areas with the same CT-SS. However, the chest CT also showed some new GGO areas in the left superior lobe (Figure 3).

The patient’s saturation level was at 97%, and her clinical condition remained stable. Thus, the patient was discharged. However, 15 days later (on 3 July), the patient returned to our emergency room for the worsening of dyspnea.

**Figure 4:**
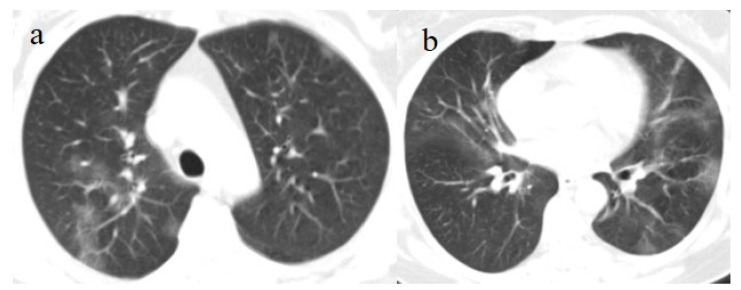
Chest CT performed on 3 July, showing in the image (**a**) that some GGO areas remained bilaterally present in the superior lobe and in the image (**b**) in the middle and inferior lobe with a multifocal distribution.

On admission, another NP/OP swab tested positive. The chest CT continued to show GGO areas, although the CT-SS remained stable (Figure 4). Treatment with ceftriaxone (1 g once daily with intravenous administration) was started, along with prednisone (25 mg once daily with oral administration for 10 days).

Her clinical conditions improved again, and the patient was discharged. However, her RT-PCR for SARS-CoV-2 remained positive.

## 4. Case #3

**Figure 5:**
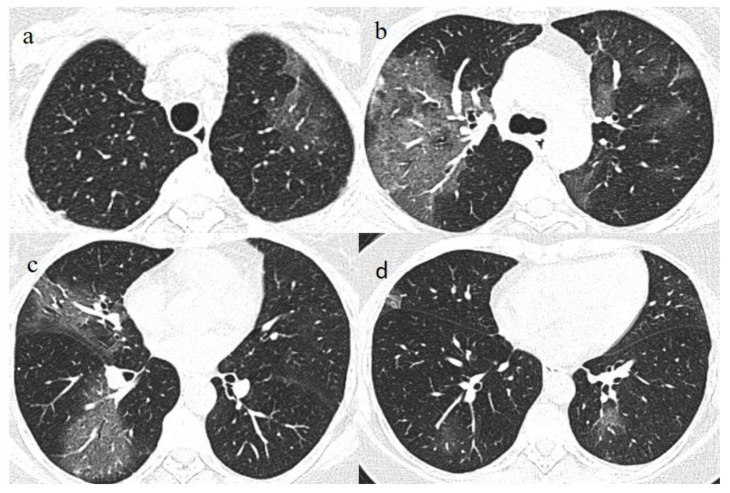
Chest CT performed in the emergency room on 1 July. (**a**) Some GGO in the left superior lobe, (**b**) in the right superior lobe, (**c**) the middle and right lobe, and (**d**) the inferior lobes.

A 53-year-old woman with a history of follicular lymphoma and undergoing treatment with rituximab was fully vaccinated with the COVID-19 mRNA-1273 Pfizer vaccine (first dose on 4 March and second dose on 26 March). About 30 days after vaccination (on 28 April), she tested positive for SARS-CoV-2, and she was also treated with antibody monoclonal therapy. After that, she continued to show three consecutive positive results for SARS-CoV-2 on RT-PCR (on 8, 16, and 20 May) and negative results on 27 May and 21 June. However, on 1 July, she visited the emergency room of our hospital with fever and dyspnea with SO2 at 90%. On admission, she tested positive for SARS-CoV-2 on the NP/OP swab without the detection of any variants. The chest CT showed GGO areas with a peripheral and central distribution and a CT-SS of 11/20 (Figure 5).

On laboratory examination, she showed a mild low-level white blood count (5100/µL), an increased value of LDH (658 IU/L), an increased level of CRP (8.50 mg/dL), and mild higher values of aspartate transaminases (AST) (55 IU/L; normal value range 5–34) and alanine transaminases (ALT) (101 IU/L; normal value range 0–55). The patient also showed a mild elevation of the D-Dimer level (1.11 mg/dL). The other laboratory values were within the normal limits. The serology, performed with an immunoassay (LiaisonXL), found only the presence of SARS-CoV-2 anti-spike IgG (200 BAU/mL), which was related to the previous vaccination.

Treatment with dexamethasone (4 mg once daily with intravenous administration for 10 days) with intravenous remdesivir (200 mg on day 1 followed by 100 mg for 4 days) and conventional oxygen therapy (1 L/min) was started with SO2 at 96%, together with LMWE 4000 IU (once daily with a subcutaneous administration for 10 days).

**Figure 6:**
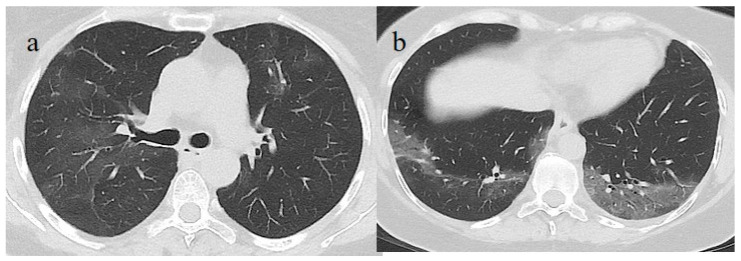
Chest CT was performed on 21 July. (**a**) The resolution of some GGO in the superior lobe. However, GGO continued to be present in the inferior lobe, as shown in (**b**).

Two days after her hospital admission, another OP/NP swab was repeated, which continued to test positive for SARS-CoV-2. However, her clinical conditions improved, and she showed two consecutive negative RT-PCR results on 9 and 13 July. Therefore, the patient was discharged. However, she returned to the emergency room of our hospital on 21 July. She tested positive Sars-CoV-2 on the OP/NP swab performed in the emergency room. The chest CT continued to show GGO, mainly in the inferior lobes with a CT-SS of 9/20 (Figure 6).

**Figure 7:**
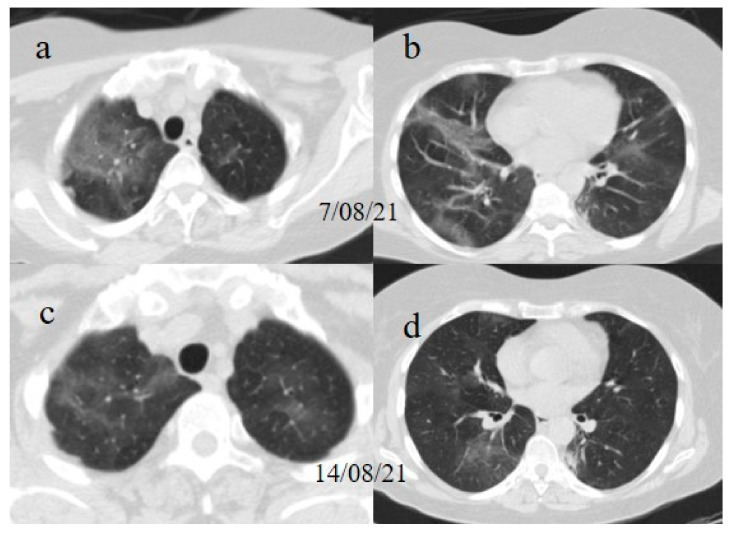
Chest CT performed on 7 August (**a**) showing some new GGO in the superior lobes and also in the middle and inferior lobes (**b**). On the chest CT performed a week later was visible the resolution of some GGO (**c**,**d**).

Treatment with meropenem (3 g/day given as three doses) and methylprednisolone (1 mg/kg daily for 3 days), followed by prednisone (25 mg once daily with oral administration for 10 days), was prescribed. The patient’s clinical conditions and dyspnea improved. However, she continued to experience fever (38.5 °C) and to test positive for SARS-CoV-2 on the OP/NP swabs on 23, 26, and 3 August with negative results on 11 August and 14 August. Another chest CT has been repeated on 7 August showing new areas of GGO, especially in the superior lobes (Figure 7). Therefore, treatment with immunoglobulin was started with a resolution of some of the previous GGO on the chest CT control made a week later on 14 August. Therefore, the patient has been discharged.

## 5. Case #4

An 80-year-old man received a full dose of the COVID-19 mRNA-1273 Pfizer vaccine (first dose on 11 March and second dose on 1 April). In addition, his wife was fully vaccinated. However, on 6 July, the patient started to experience cough, dyspnea, and fever.

Seven days later, his clinical condition worsened, and he visited the emergency room of our hospital. His fever was very high (39.5 °C).

On admission, an NP/OP swab tested positive for SARS-CoV-2. The patient denied any contact with individuals who had tested positive for COVID-19. No Delta variant was detected. On laboratory examination, he showed mild lymphopenia (830/µL; normal value range 800–5000/µL) and a mild lower hemoglobin value (11.1 mg/dL). The other laboratory values were within the normal ranges.

On admission, the SO2 was at 89% with PO2/FiO2 300.

**Figure 8:**
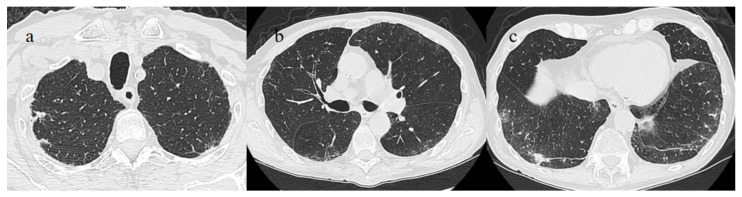
Chest CT performed 13 July showing some small consolidation areas with a peripheral and posterior distribution in the superior lobes (**a**), part of the superior ad inferior lobes (**b**) and at the basal level of the inferior lobes (**c**).

A chest CT showed some small consolidation areas in a peripheral posterior distribution typical of COVID-19 pneumonia, with a CT-SS score of 5/20 (Figure 8).

The serology, performed with an immunoassay (LiaisonXL), showed the absence of SARS-CoV-22 S1/S2 IgM and the presence of SARS-CoV-2 anti-spike IgG (322 BAU/mL) related to the previous vaccination.

Treatment with dexamethasone (6 mg once daily with intravenous administration for 10 days) and conventional oxygen therapy was started, together with LMWE 2000 IU (once daily with a subcutaneous administration for 10 days).

Another NP/OP swab was repeated on 16 July, and the patient continued to test positive for SARS-CoV-2. On 16 July, SO2 was at 95%, PO2/FiO2 320.

However, the patient’s clinical condition remained stable.

## 6. Case #5

**Figure 9:**
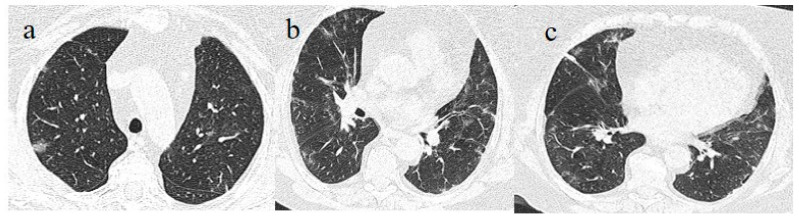
Chest CT performed on 21 July showing some central and peripheral GGO with interstitial thickness and multifocal distribution in the superior lobes (**a**), in the middle and inferior lobes (**b**) and in the inferior lobes (**c**).

A 63-year-old woman visited the emergency department of our hospital on 30 July with fever and dyspnea with also thoracic pain. She had a history of previous uterine cancer treated with surgery 10 years before. She had already tested positive for SARS-CoV-2 on 21 July 2021 on an OP/NP swab conducted by an authorized laboratory. She was also fully vaccinated with the COVID-19 mRNA-1273 Pfizer vaccine (first dose on 22 April and second dose on 14 May). She was treated with an oral administration of azithromycin (500 mg 1 cp once daily) for 3 days by her family doctor. However, 2 days before hospital admission, she reported nausea and vomiting. At the time of hospital admission, an NP/OP swab tested positive for SARS-CoV-2, however, without the detection of any variants. The woman reported that her relatives, including her husband and daughter, were also fully vaccinated against COVID-19, and they had also tested positive for SARS-CoV-2. Her daughter reported recent contact with her boyfriend, who had also tested positive for SARS-CoV-2 prior to the daughter’s COVID-19 infection. The daughter’s boyfriend was also fully vaccinated. On hospital admission, during laboratory examination, the woman showed mild elevations in CRP (3.98 mg/dL), LDH (309 IU/L), and the D-dimer (0.51 mg/L). The other laboratory values were within the normal range. Her SO2 level was at 94%. However, due to also to the history of previous uterine cancer and the presence of dyspnea a chest CT was made in an emergency. On the chest CT, some GGO areas were found in a peripheral distribution, suggesting COVID-19 pneumonia with a CT-SS of 5/20 (Figure 9). No serology for SARS-CoV-2 was performed. Treatment with ceftriaxone (1 g once daily with intravenous administration) was started, together with dexamethasone (4 mg once daily with intravenous administration) and LMWE 4000 IU (once daily with a subcutaneous administration). The patient’s clinical conditions improved, and she was discharged after 3 days.

## 7. Case #6

A 61-year-old man received a full dose of the COVID-19 mRNA-1273 Pfizer vaccine (first dose on 22 April and second dose on 14 May). The patient had a history of hyperintensive cardiopathy and a previous cerebral hemorrhage (in 2018). However, on 7 July, he came to the emergency department of our hospital for a lipothymic episode. He did not present a confused state, and he also reported chest pain. He did not report a history of fever.

However, on admission, an NP/OP swab tested positive for SARS-CoV-2 without the detection of any variants. The patient denied any contact with individuals who had tested positive for COVID-19.

**Figure 10:**
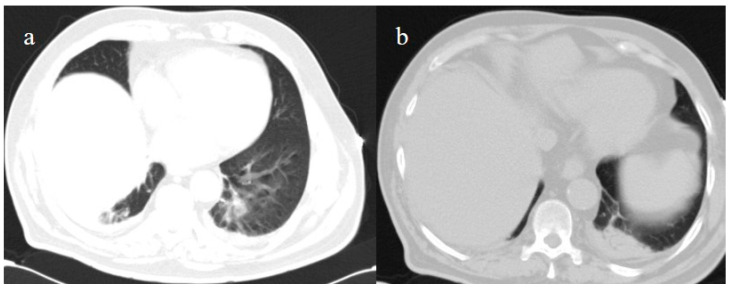
Some GGO and consolidation located at the peripheral and posterior inferior lobes, with some consolidation and small pleural effusion (**a**,**b**) were incidentally found on the chest CT performed on 7 July for thoracic pain in a patient who tested positive for SARS-CoV-2 and who came to the emergency room for a lipothymic episode.

On laboratory examination, the patient showed a mild low level of hemoglobin (Hb, 12 g/dL), a low level of platelet counts (129.000/µL; normal values 150.000–400.000/µL), a mild elevation of D-Dimer levels (1.28 mg/L), and a mild elevation of CRP (3.02 mg/dL). The other laboratory values were within the normal limits. His SO2 level was at 95%. Brain and chest CT scans were performed in the emergency department. No acute lesions were detected on the brain CT, but some GGO with consolidation areas were found on the chest CT on the left inferior lobes with a mild pleural effusion (Figure 10). Treatment with prednisone (25 mg once daily with oral administration for 10 days) was started, together with LMWE 4000 IU (once daily with a subcutaneous administration for 10 days). However, the patient’s OP/NP continued to test positive on 18 and 21 of July, but the patient’s clinical conditions remained stable. Thus, the patient was discharged.

## 8. Discussion

The approved vaccines developed by Pfizer and Moderna use mRNA technology, while the approved formulations by AstraZeneca and Johnson and Johnson contain DNA delivered within non-replicating recombinant adenovirus (AdV) vector systems [24]. These COVID-19 vaccines have demonstrated high efficacy at preventing symptomatic disease, as shown by clinical trials [15,16]. The effectiveness of the BNT162b2 vaccine in reducing infection, severe disease, hospitalization, and death with COVID-19 patients has been reported in Israel [16,25]. The ChAdOx1 nCoV-19 trial found an efficacy against symptomatic disease of 76% at 22–90 days after at least one standard dose [15,26,27]. The efficacy of the vaccine further increases after the second dose [16,28].

However, some genetic variants, such as the Delta variant, have emerged more recently. To date, only limited data on these variants are available in vaccinated people. Emerging studies have shown that two-dose administration resulted in efficacy against variants, such as the Delta variant [28,29]. In contrast, one dose was not shown to be protective [30]. Indeed, Williams et al. [30] reported that 8/21 residents and 14/21 staff members that had previously received a single dose of the Vaxzevria vaccine were infected by the Delta variant, but none of them died. On the other hand, in our cases, the only patient that died was affected by COVID-19 pneumonia related to the Delta variant.

However, our patient was older than the cases described by Williams et al. [30] and he manifested the first symptoms related to Covid-19 infection three days after the second dose of vaccination, in a not fully protective state. Indeed, the protective effect of the second dose vaccines is usually observed after 14 days of vaccination, as has been reported in the literature [31]. For this reason, case 1 may have been infected by SARS-CoV-2 some days after the vaccination

However, COVID-19 pneumonia has been rarely described in fully vaccinated patients and there are no yet ongoing clinical trials in these people categories. Surprisingly, so far, we have found only two described clinical cases [32,33].

All our cases after available SARS-CoV-2 2 vaccination presented with clinical and radiological findings of typical COVID-19 with different grading of severity infection, probably related to timing and different response to vaccination. In three patients (cases 4–6), we found only mild COVID-19 pneumonia. Our results show that vaccination can be protective against the aggressive form of COVID-19 pneumonia. In fact, in our case 4, a fully vaccinated old patient developed only mild COVID-19 pneumonia. In case 6, the detection of RT-PCR positivity for SARS-CoV-2 and of GGO on the chest CT scan were not expected and interpreted as an incidental finding because the patient came to the emergency room for a lipothymic episode that is not a typical finding of COVID-19 pneumonia presentation. The patient did not present fever or dyspnea. However syncopal or pre-syncopal episodes have been also reported as the only initial symptoms of COVID-19 infection [34].

We should highlight that pneumonia also in mild presentation has been not yet reported in fully vaccinated patients and our 5 cases have been not detected any variants but only in one case.

Therefore, full vaccination is important to protect individuals from severe disease.

However, mild symptoms with RT-PCR positivity have also been reported in fully vaccinated patients, suggesting that patients, even if asymptomatic, may still be become infected and transmit the live virus from the upper airway [35,36]. In fact, in our case 5, the patient has been infected by her full vaccinated relatives.

Therefore, the use of masking and social distancing should be also continued in vaccinated people especially in all closed environments [36] and isolation recommendations should be considered even for the fully vaccinated if in contact with suspected cases of COVID-19.

In addition, our reports raised the question related to the oncologic patient that became affected by COVID-19 even if in presence of vaccination. Two patients among our cases affected by lymphoma treated with rituximab therapy presented with COVID-19 pneumonia infection after vaccination: in case 2, SARS-CoV-2 serology was not detected whereas in case 3, only the presence of SARS-CoV-2 anti-spike IgG was detected.

These data suggested that is also important to monitor the typical clinical status related to oncologic patients. Indeed, an immunocompromised state and the age-related decline of the immune response can compromise the vaccination efficacy [31,37].

It has been also reported that the use of the anti-CD20 antibody rituximab, with or without chemotherapy, is typically associated with impaired humoral responses to influenza vaccines [38]. Similar cases are emerging with SARS-CoV-2 vaccines [39,40,41,42,43]. Rituximab is associated with B cell 141 depletion for up to 78 weeks following use. On the other hand, patients with non-Hodgkin lymphomas (NHLs) are associated with disease-related immunodeficiency, which may render these patients especially susceptible to SARS-CoV-2 infection [39]. Immunocompromised patients likely acquire different clades of SARS-CoV-2 45 infection. Moreover, they cannot respond to the Sars-Cov-2 vaccines [42,43]. Alshukairi et al. [42] recently described the case of a 51-year-old female patient with a history of follicular non-Hodgkin’s lymphoma and rituximab therapy. The patient did not develop immunity after SARS-CoV-2 infection and did not respond to the mRNA COVID-19 vaccine. This case is very similar to two of our reported cases. However, in our two cases, the patients continued to test positive for SARS-CoV-2. Patients in an immunocompromised state should be always protected primarily in their own environment (e.g., hospitals and wards where this population meets there should be constantly testing to avoid inter-patient transmission).

Therefore, it is important to also consider the serology after the COVID-19 vaccination. Not all people, especially those that are immunocompromised, develop a protective immune response after vaccination [31,37,41]. On the other hand, we should also consider that the age-related decline in immunity can reduce the prophylactic efficacy of vaccinations. Muller et al. [44] reported that 31.3% of the study’s elderly subjects had no detectable neutralizing antibodies after vaccination in contrast to the younger subjects. Therefore, to reduce virus transmission, we believe that it is important to continue applying the same rules, such as the use of masks, to all people, including vaccinated people. We also recommend individuating the rapid tracking of the virus with RT-PCR in mild symptomatic vaccinated patients and monitoring the immune response through the serology state, as not all people respond to the SARS-CoV-2 vaccination. In addition, the development of quicker RT-PCR for SARS-CoV-2 with major sensitivity and specificity can be useful to fight and contain the pandemic [45]. Brotons et al. [45], proposed a direct RT-PCR on self-collected raw saliva as a rapid method of screening, with a sensitivity and specificity, respectively, of 95.7% and 100.0%. We believe that caution should be preserved because some factors can influence vaccine immunogenicity as age factors and the presence of an immunocompromised state. On the other end, emergent researches also raised the question to consider the possibility of Acute enhancement disease (ADE) in presence of virus variants even if this phenomenon has been not fully demonstrated in the clinical trials [31,46]. Future clinical trials and studies in vitro and animal models should be done to better understand this mechanism.

## 9. Conclusions

After the establishment of the vaccine as the primary and most efficient tool of counter fighting the pandemic, it is expected not to offer 100% protection. Our cases highlight that fully vaccinated patients can also be affected by COVID-19 pneumonia in a mild form. Not all people develop an effective immune response, and an individual’s immune response depends on their immunocompromised state. Therefore, monitoring the serology state, as well as the use of masks and social distancing, should be continued in environments with high percentages of vaccinated people.

The continuous updating of COVID-19 vaccines should also be considered [47]. Furthermore, further clinical trials should be done to better understand how various factors can influence vaccine immunogenicity as the presence of virus mutations, age factors, and the presence of an immunocompromised state.

## Data Availability

Data sharing is not applicable.
